# Single‐Cell Profiling and Proteomics‐Based Insights Into mTORC1‐Mediated Angio+TAMs Polarization in Recurrent IDH‐Mutant Gliomas

**DOI:** 10.1111/cns.70371

**Published:** 2025-04-09

**Authors:** Xu Wang, Jingyan Gu, Hongyu Tang, Lianping Gu, Yunke Bi, Yue Kong, Qiao Shan, Jian Yin, Meiqing Lou, Shouwei Li, Yaohua Liu

**Affiliations:** ^1^ Department of Neurosurgery Shanghai General Hospital Shanghai China; ^2^ Shanghai Jiao Tong University School of Medicine Shanghai China; ^3^ Nanjing Medical University Jiangsu China; ^4^ Sun Yat‐Sen University Guanghua School of Stomatology Guangzhou China; ^5^ Department of Neurosurgery Capital Medical University Sanbo Brain Hospital Beijing China

**Keywords:** IDH‐mutant gliomas, mTORC1 pathway, phosphoproteomics, proteomics, single‐cell sequencing, spatial transcriptomics, tumor‐associated macrophages

## Abstract

**Background:**

IDH mutant gliomas often exhibit recurrence and progression, with the mTORC1 pathway and tumor‐associated macrophages potentially contributing to these processes. However, the precise mechanisms are not fully understood. This study seeks to investigate these relationships using proteomic, phosphoproteomic, and multi‐dimensional transcriptomic approaches.

**Methods:**

This study established a matched transcriptomic, proteomic, and phosphoproteomic cohort of IDH‐mutant gliomas with recurrence and progression, incorporating multiple glioma‐related datasets. We first identified the genomic landscape of recurrent IDH‐mutant gliomas through multi‐dimensional differential enrichment, GSVA, and deconvolution analyses. Next, we explored tumor‐associated macrophage subpopulations using single‐cell sequencing in mouse models of IDH‐mutant and wild‐type gliomas, analyzing transcriptional changes via AddmodelScore and pseudotime analysis. We then identified these subpopulations in matched primary and recurrent IDH‐mutant datasets, investigating their interactions with the tumor microenvironment and performing deconvolution to explore their contribution to glioma progression. Finally, spatial transcriptomics was used to map these subpopulations to glioma tissue sections, revealing spatial co‐localization with mTORC1 and angiogenesis‐related pathways.

**Results:**

Multi‐dimensional differential enrichment, GSVA, and deconvolution analyses indicated that the mTORC1 pathway and the proportion of M2 macrophages are upregulated during the recurrence and progression of IDH‐mutant gliomas. CGGA database analysis showed that mTORC1 activity is significantly higher in recurrent IDH‐mutant gliomas compared to IDH‐wildtype, with a correlation to M2 macrophage infiltration. KSEA revealed that AURKA is enriched during progression, and its inhibition reduces mTORC1 pathway activity. Single‐cell sequencing in mouse models identified a distinct glioma subpopulation with upregulated mTORC1, exhibiting both M2 macrophage and angiogenesis transcriptional features, which increased after implantation of IDH‐mutant tumor cells. Similarly, human glioma single‐cell data revealed the same subpopulation, with cell–cell communication analysis showing active VEGF signaling. Finally, spatial transcriptomics deconvolution confirmed the co‐localization of this subpopulation with mTORC1 and VEGFA in high‐grade IDH‐mutant gliomas.

**Conclusions:**

Our findings suggest mTORC1 activation and Angio‐TAMs play key roles in the recurrence and progression of IDH‐mutant gliomas.

## Graphical abstract

mTORC1 signaling drives the polarization of Angio+TAMs in recurrent IDH‐mutant gliomas, promoting angiogenesis and immune evasion, which contributes to tumor progression and recurrence in the glioma microenvironment.

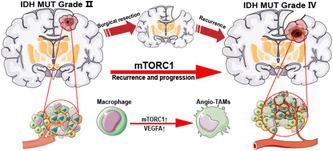

## 1 Introduction

Gliomas pose significant challenges due to their highly aggressive nature. Despite the implementation of multidisciplinary treatment approaches, patient survival rates remain disappointingly low [1, 2]. The inherent malignancy of gliomas, coupled with their propensity for invasion and recurrence, necessitates ongoing research into more effective therapeutic strategies.

The 2021 classification of gliomas by the World Health Organization (WHO) has provided crucial insights into their molecular characteristics [3]. Notably, the presence or absence of isocitrate dehydrogenase (IDH) mutations has emerged as a pivotal factor in distinguishing between glioblastoma (GBM) and astrocytomas. Tumors harboring IDH mutations generally exhibit a more favorable prognosis, yet a subset of astrocytomas continues to exhibit recurrence and disease progression despite initial favorable outcomes. This highlights the need for a more nuanced understanding of the underlying mechanisms driving glioma heterogeneity.

The mechanistic target of rapamycin complex 1 (mTORC1) regulates key cellular processes, such as growth, survival, apoptosis, and angiogenesis [4]. Comprising mTOR, mLST8, RAPTOR, and two negative regulators, PRAS40 and Deptor, mTORC1 is sensitive to rapamycin and plays a pivotal role in tumor biology [5]. Recent studies have highlighted the activation of mTORC1 in IDH mutant gliomas, suggesting a significant involvement of this pathway in gliomagenesis [6]. Clinical trials have demonstrated that oral administration of mTORC1 inhibitors can effectively reduce angiogenesis in IDH‐mutant low‐grade gliomas, supporting the therapeutic potential of targeting this pathway [7]. However, the precise mechanisms by which mTORC1 contributes to the initiation and progression of gliomas remain to be elucidated. Understanding these mechanisms could reveal novel therapeutic targets and inform the development of more effective treatment strategies for patients with gliomas.

Moreover, the pathogenesis of gliomas is intricately linked to a complex immune microenvironment, which remains poorly understood. Tumor‐associated macrophages (TAMs) represent a critical component in the tumor microenvironment of gliomas, playing a pivotal role in tumor growth, invasion, metastasis, and angiogenesis [8, 9, 10]. Despite their significance, the precise molecular mechanisms underlying TAM function in gliomas remain inadequately understood. Recent studies show that TAM composition in IDH‐mutant gliomas differs from that in wild‐type gliomas [11]. Notably, TAMs in IDH‐mutant gliomas predominantly exhibit an unactivated, immunosuppressive phenotype [12]. This immune evasion may contribute to the distinct biological behavior of IDH‐mutant gliomas. Furthermore, mTORC1 has been shown to exert differential regulatory effects on TAMs derived from various tumor types [13, 14]. However, the exact pathways and interactions involved in this modulation remain to be elucidated. In conclusion, elucidating the multifaceted roles of TAMs in gliomas, particularly in the context of IDH mutations and mTORC1 signaling, is crucial for developing innovative therapeutic approaches.

Our study utilized matched samples of IDH‐mutant gliomas that exhibited recurrence and progression to perform transcriptomic, proteomic, and phosphoproteomic sequencing. We combined these findings with single‐cell sequencing and spatial transcriptomics from public databases to investigate potential targets involved in the recurrence progression of IDH‐mutant gliomas and to explore the impact of mTORC1 activation on the immune microenvironment of these tumors. We focused particularly on changes in the immune microenvironment associated with gliomas. Our results indicate that in IDH‐mutant gliomas, mTORC1 is activated by AURKA, promoting the phenotypic transformation of macrophages and facilitating angiogenesis, which ultimately contributes to the recurrence progression of IDH‐mutant gliomas.

## 2 Materials and Methods

### 2.1 Astrocytoma Cohort Collection and Preparation

We established a cohort named SHGH‐PR‐A, which includes a total of 16 matched glioma tumor samples obtained from 8 patients who underwent resection of brain glioma lesions at Shanghai General Hospital. According to the 2021 WHO classification of gliomas, the initial pathological diagnosis of these patients was WHO grade 2 IDH‐mutant astrocytoma, and the pathological diagnosis following recurrence was WHO grade 4 IDH‐mutant astrocytoma. This study was approved by the Ethics Committee of Shanghai General Hospital (2021SQ054). Detailed protocols for glioma tissue preparation used in proteomics and phosphoproteomics studies are provided in the Appendix S1.

### 2.2 Public Data Acquisition and Sample Grouping

The mRNA‐seq‐693 dataset from the Chinese Glioma Genome Atlas (CGGA) database (http://www.cgga.org.cn) was used, which includes data from 443 lower‐grade gliomas (LGG) and 249 GBM [15]. This dataset served as an external validation cohort, which included primary IDH‐mutant astrocytoma samples (‘pA’) and recurrent IDH‐mutant GBM samples (‘rA’), totaling 78 samples. Similarly, 25 samples each from primary and recurrent IDH wild‐type GBM were randomly selected, yielding 128 glioma samples. The dataset includes RNA sequencing data, WHO grading, pathological features, and survival information. Additionally, the following datasets were utilized for analysis: GSE152612 (Bulk RNA‐Seq), GSM6226123 (single‐cell RNA‐Seq), and GSE166218 (single‐cell RNA‐Seq), all obtained from the Gene Expression Omnibus (GEO) database (https://www.ncbi.nlm.nih.gov/) [16]. The GSE152612 dataset (platform: GPL17930) comprises data from four groups of GL261 mouse glioma cells, with two groups treated with AURKA inhibition and two control groups [17]. The GSM6226123 dataset (platform: GPL24676) includes single‐cell RNA sequencing data from 12 matched primary and recurrent IDH‐mutant glioma patients, which were used for subsequent analyses [18]. The GSE166218 dataset (platform: GPL21103) includes sequencing data from CD45^+^ cells from four groups of GL261 tumor‐bearing mice, with wild‐type and IDH1‐R132H mutant GL261 cells implanted intracranially. Mice were euthanized at 7 or 28 days post‐implantation, and CD45^+^ cells were subsequently sorted for analysis [19]. We downloaded the spatial transcriptomic data from Dryad (https://datadryad.org/stash/dataset/10.5061/dryad. h70rxwdmj) and GSE237183 from the GEO database. Lastly, 200 mTORC1 signaling pathway genes were retrieved from the Human MSigDB Collections database (https://www.gsea‐msigdb.org/gsea/msigdb/collections.jsp) for further analysis [20].

### 2.3 Differential Expression Analysis, Enrichment Analysis, and GSEA Analysis

Differential expression analysis of transcriptomic, proteomic, and phosphoproteomic data was performed using the limma package to compare primary and recurrent glioma samples within the same IDH status [21]. This analysis aimed to identify differentially expressed genes (DEGs), proteins (DEPs), and phosphorylated sites (DEPPs), with significance thresholds set at *p* < 0.05 and |log2FoldChange (log2FC)| > 1. The resulting differential expression sets were visualized using volcano plots. To further explore the biological functions of the differentially regulated genes, gene enrichment analysis was conducted on the upregulated and downregulated gene groups using the clusterProfiler package, and the results were visualized with the ggplot2 package [22]. The expression patterns of the Hallmark mTORC1 signaling pathway genes across transcriptomic, proteomic, and phosphoproteomic datasets were visualized using heatmaps generated by the ComplexHeatmap package. Additionally, to investigate the biological functional differences between primary and recurrent IDH‐mutant gliomas, the h.all.v2023.1.Hs.symbols.gmt gene set from the MSigDB was used as a reference [20]. Differential expression analysis was conducted on the CGGA counts dataset using the DESeq2 package to calculate log2FC and rank genes by magnitude in descending order. Gene set enrichment analysis (GSEA) was then performed using the clusterProfiler package to identify enriched biological pathways.

### 2.4 GSVA and Survival Analysis

Functional scores for glioma samples and cellular subpopulations from the CGGA dataset, GSE152612, and single‐cell data were calculated using the gene set variation analysis (GSVA) algorithm in the GSVA package, based on multiple gene sets from the Hallmark database [23]. The mTORC1 scores in different subgroups of the CGGA dataset were visualized and compared using boxplots generated by the ggplot2 package. Pathway scores for multiple pathways in the GSE152612 dataset were visualized through heatmaps created by the ComplexHeatmap package. For the CGGA dataset, samples were first divided into two subgroups based on their IDH status, and then within each subgroup, patients were categorized into high‐ and low‐score groups based on the median mTORC1 score. Kaplan–Meier (K‐M) survival analysis was performed using the survminer package to evaluate the impact of mTORC1 scores on the survival rates of patients in the two subgroups.

### 2.5 CIBERSORT Analysis and Correlation Analysis

Deconvolution analysis of immune infiltration was performed using the CIBERSORT algorithm, with gene expression profiles of 22 immune cell types obtained from the CIBERSORTX website (https://cibersortx.stanford.edu/) [24]. This analysis was applied to both the self‐collected and CGGA datasets to estimate immune cell infiltration in each sample. Spearman correlation analysis was conducted using the Corrplot package to assess the relationship between the proportion of M2 macrophages and mTORC1 pathway scores as well as AURKA expression levels in different subgroups. The results were visually represented using scatter plots for clarity.

### 2.6 KSEA Analysis

To explore the kinase‐enriched phosphorylated sites before and after the progression of IDH‐mutant glioma recurrence, human kinase‐substrate annotations were extracted from the PhosphoSitePlus database [25]. These annotations were then mapped to our phosphoproteomic data by matching with phosphorylation sites in the UniProt database. Subsequently, kinase‐substrate enrichment analysis (KSEA) was performed using the ksea package in R. Kinases associated with fewer than 10 quantified substrates in a given model were considered insignificant and excluded from the analysis. A total of 1000 permutations were executed to calculate the empirical *p*‐values.

### 2.7 Single‐Cell Data Dimensionality Reduction, Clustering, Visualization, and Cell Annotation

Single‐cell RNA sequencing (scRNA‐seq) analysis was performed on the GSM6226123 and GSE166218 datasets to investigate the distribution of the mTORC1 pathway at the cellular level. Quality control (QC) analysis was first conducted to remove low‐quality samples, and Seurat objects were created using the Seurat package (min. cells = 3; min. features = 100). Doublet detection and removal were carried out using the DoubletFinder package, and cells with mitochondrial content below 10% were excluded [25]. Batch effects were removed using the FastMNN method. Following QC, the FindVariableFeatures function was used to identify highly variable genes. Principal component analysis (PCA) was then performed based on the highly variable genes to assess cell distribution and identify outliers. The percentage of variance explained by each principal component (PC) was evaluated, and a PCA elbow plot was generated to select the PCs before the elbow for subsequent analysis. Cell clustering was performed using the Seurat package with the FindNeighbors and FindClusters functions for dimensionality reduction. The results were visualized using uniform manifold approximation and projection(UMAP) and t‐distributed stochastic neighbor embedding(t‐SNE) algorithms. Additionally, cell types were annotated based on marker genes from prior studies, and a dot plot was generated to show the expression of marker genes across different cell types [26]. For subgroup analysis, glioma tumor cell subpopulations were classified into MES, NPC, OPC, and AC groups based on gene set scoring using GSVA and literature‐based marker gene sets [27]. The FindAllMarkers function was utilized to identify signature marker genes for macrophage subgroup classification.

### 2.8 AddModelScore and mTORC1 Macrophage Function Visualization

The mTORC1 signature scores for different cells were calculated using the AddModuleScore function in Seurat [28]. This function computes the average expression of a given feature in each cell and subtracts the average expression of randomly selected control features. The calculation was performed using 100 control features. The mTORC1 scores between different cell subpopulations were visualized using violin plots. Since the scores follow a normal distribution, two‐sample *t*‐tests were used to compare significance between the subpopulations. To compare the distribution of mTORC1 in TAMs, t‐SNE was employed to visualize mTORC1 scores in TAMs. Based on the median score, TAMs were categorized into two subgroups: mTORC1_High and mTORC1_Low. The differential distribution of genes related to angiogenesis and M2 polarization pathways between these two subgroups was visualized using heatmaps generated by the ComplexHeatmap package.

### 2.9 Pseudotime and CellChat Analysis

Based on the GSE166218 dataset, macrophages from mice were extracted for pseudotime analysis. The Monocle 2 algorithm was used to construct a single‐cell trajectory plot, projecting all cells from the single‐cell clusters onto the roots and branches [29]. The expression changes of the mTORC1 pathway, angiogenesis, and M2 marker genes were then assessed along the pseudotime. The CellChat tool was employed to quantify receptor‐ligand interactions between different cell types in the GSM6226123 dataset, aiming to infer intercellular communication networks [30]. Heatmaps were generated using the pheatmap package to visualize interaction pathways specific to each cell type.

### 2.10 Spatial Transcriptomics, Spotlight Deconvolution, and Spatial Cell Communication Analysis

Spatial transcriptomics data constructed using 10X Visium were analyzed with the Seurat package in R. Cross‐point normalization was performed using the “SCTransform” function. PCA for dimensionality reduction and clustering was carried out using the “RunPCA” function. The “GSVA” package was used to score each SPOT based on gene sets related to the mTORC1 pathway. The “SPOTlight” package, employing non‐negative matrix factorization (NMF), was used to deconvolve cell subtype distributions in the spatial transcriptomics data, leveraging prior analysis from the GSM6226123 scRNA‐seq dataset [31]. Correlation analysis between mTORC1 pathway scores and the proportion of Mo‐C2 subpopulations across different IDH mutation levels in glioma was performed using the Corrplot package.

### 2.11 Statistical Analysis

Data processing and analysis were conducted using R software. Given the non‐normal distribution of multi‐omics data, such as RNA sequencing, proteomics, and phosphoproteomics. For RNA sequencing data, Limma was used, which is suitable for count‐based data. For other datasets, non‐parametric statistical tests, including the Wilcoxon rank‐sum test, were applied to assess group differences. Statistical significance was defined as a *p*‐value less than 0.05.

## 3 Results

### 3.1 Matched Transcriptomic, Proteomic, and Phosphoproteomic Profiling Reveals Consistent Molecular Changes in IDH‐Mutant Gliomas During Recurrence

**FIGURE 1:**
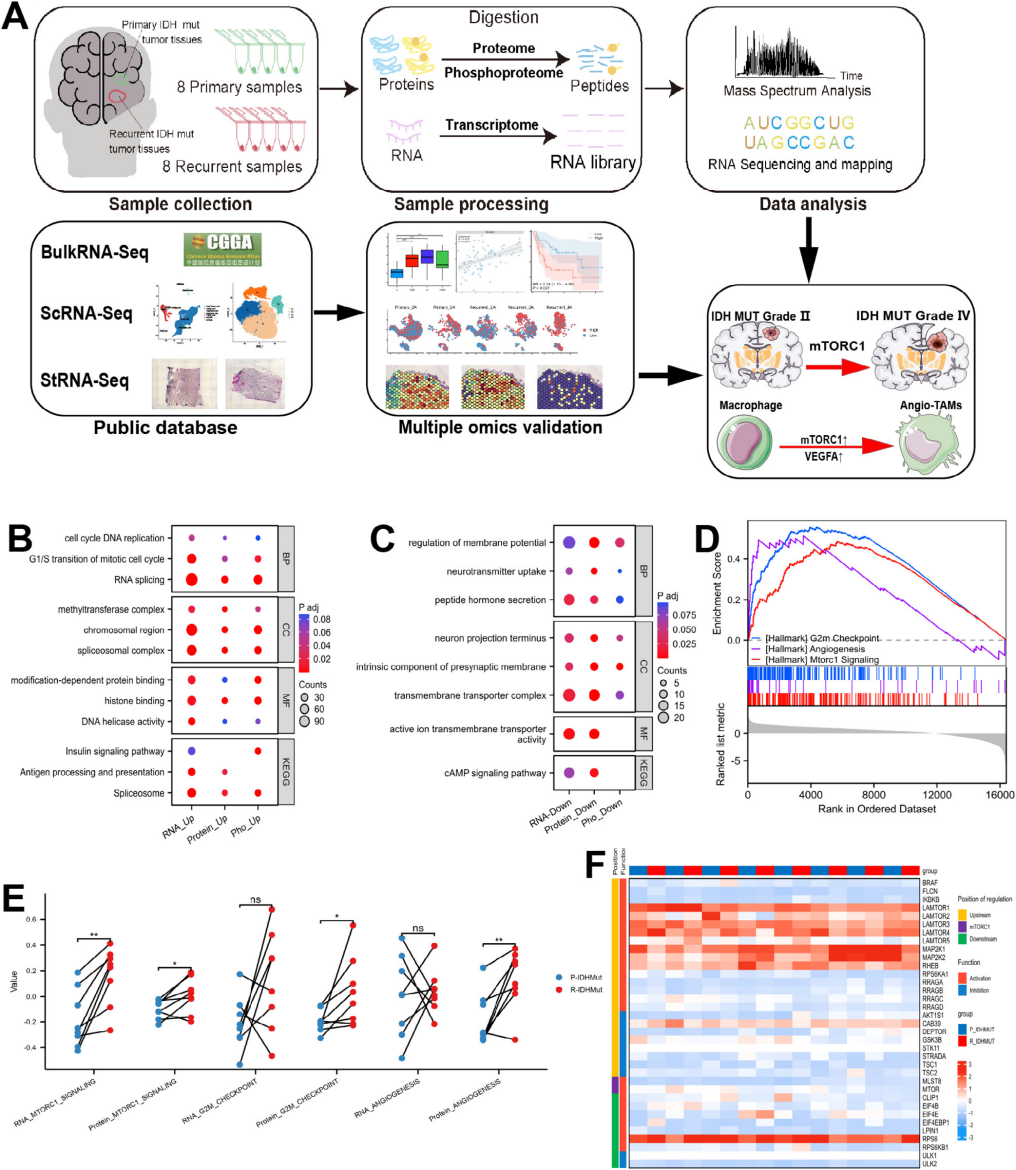
Omics landscape of primary and recurrent IDH‐mutant gliomas. (A) Schematic overview of the experimental design. (B) Enrichment analysis of upregulated molecules from multi‐dimensional transcriptomic differential analyses. (C) Enrichment analysis of downregulated molecules from multi‐dimensional transcriptomic differential analyses. (D) GSEA analysis of upregulated pathways following recurrence. (E) Comparing GSVA scores of mTORC1, angiogenesis, and G2M pathways in primary vs. recurrent IDH‐mutant gliomas. (F) Heatmap of key mTORC1 pathway molecules in the proteomics levels. *p < 0.05, **p < 0.01.

To comprehensively investigate the transcriptomic, proteomic, and phosphoproteomic changes in IDH‐mutant gliomas at both initial and recurrent stages, we collected 16 matched samples from 8 patients at the Department of Neurosurgery, Shanghai General Hospital, including primary and recurrent IDH‐mutant gliomas. These samples underwent systematic analyses utilizing transcriptomics, proteomics, and phosphoproteomics (Figure 1A).

Our transcriptomic analysis identified a total of 46,833 RNA species in the matched IDH‐mutant glioma samples, while the proteomic analysis detected 8821 proteins, and the phosphoproteomic analysis revealed 41,917 phosphorylation sites across 6523 proteins. Differentially expressed gene (DEG) analysis indicated that, compared to paired initial samples, recurrent samples exhibited 2274 upregulated and 665 downregulated genes (log2FC > 1, *p* < 0.05) (Figure S1A, Table S1). Similarly, differential expression protein (DEP) analysis identified 314 upregulated and 201 downregulated proteins in recurrent samples compared to initial samples (log2FC > 1, *p* < 0.05) (Figure S1B, Table S2). Furthermore, the analysis of differential phosphorylation sites revealed 800 upregulated and 272 downregulated sites in recurrent samples (log2FC > 1, *p* < 0.05) (Figure S1C, Table S3). We found considerable discrepancies between the differential proteins and differential phosphorylation sites in the recurrent glioma group (Figure S1D).

To explore the concordant changes in RNA, proteins, and phosphorylation processes during the recurrence of IDH‐mutant gliomas, we performed gene set enrichment analysis on the differentially expressed genes, proteins, and their corresponding phosphorylated proteins. The results of Gene Ontology (GO) and Kyoto Encyclopedia of Genes and Genomes (KEGG) enrichment analyses indicated that pathways related to RNA splicing, cell cycle, insulin signaling, and antibody production and secretion were upregulated during the recurrence stage of IDH‐mutant gliomas. In contrast, pathways involving membrane protein regulation, peptide hormone secretion, and transport complexes were downregulated (Figure 1B,C). Additionally, Hallmark GSEA showed significant upregulation of the G2M, angiogenesis, and mTORC1 signaling pathways in the recurrent group (Figure 1D). Given that mTORC1 is considered to be associated with splicing in various tumors, we further investigated its role in gliomas.

### 3.2 Key Role of the mTORC1 Pathway in the Recurrence of IDH‐Mutant Gliomas

Considering the pivotal role of mTORC1 in various tumors, we conducted GSVA on our data alongside data from the CGGA database. The results revealed that in matched IDH‐mutant primary and recurrent gliomas, recurrent gliomas exhibited significant activation of the mTORC1 pathway at both RNA and protein levels, achieving statistical significance. Additionally, pathways related to angiogenesis and the G2M checkpoint were also upregulated at the protein level (Figure 1E, Table S4). Our multi‐omics cohort data enabled us to construct heatmaps representing the mTORC1 pathway at transcriptional, protein, and phosphorylation levels (Figure 1F, Figure S1E,F). We observed the upregulation of several key upstream and downstream molecules associated with the mTORC1 pathway, such as LAMTOR5, EIF4E, EIF4B, and RPS6, at transcriptional, protein, and phosphorylation levels. Our research has also uncovered the upregulation of phosphorylation at specific sites during glioma recurrence, notably at Thr70 and Thr46 of EIF4EBP1, and Tyr705 of STAT3. This series of changes indicates that the mTORC1 pathway may play a crucial role in recurrent IDH‐mutant gliomas.

**FIGURE 2:**
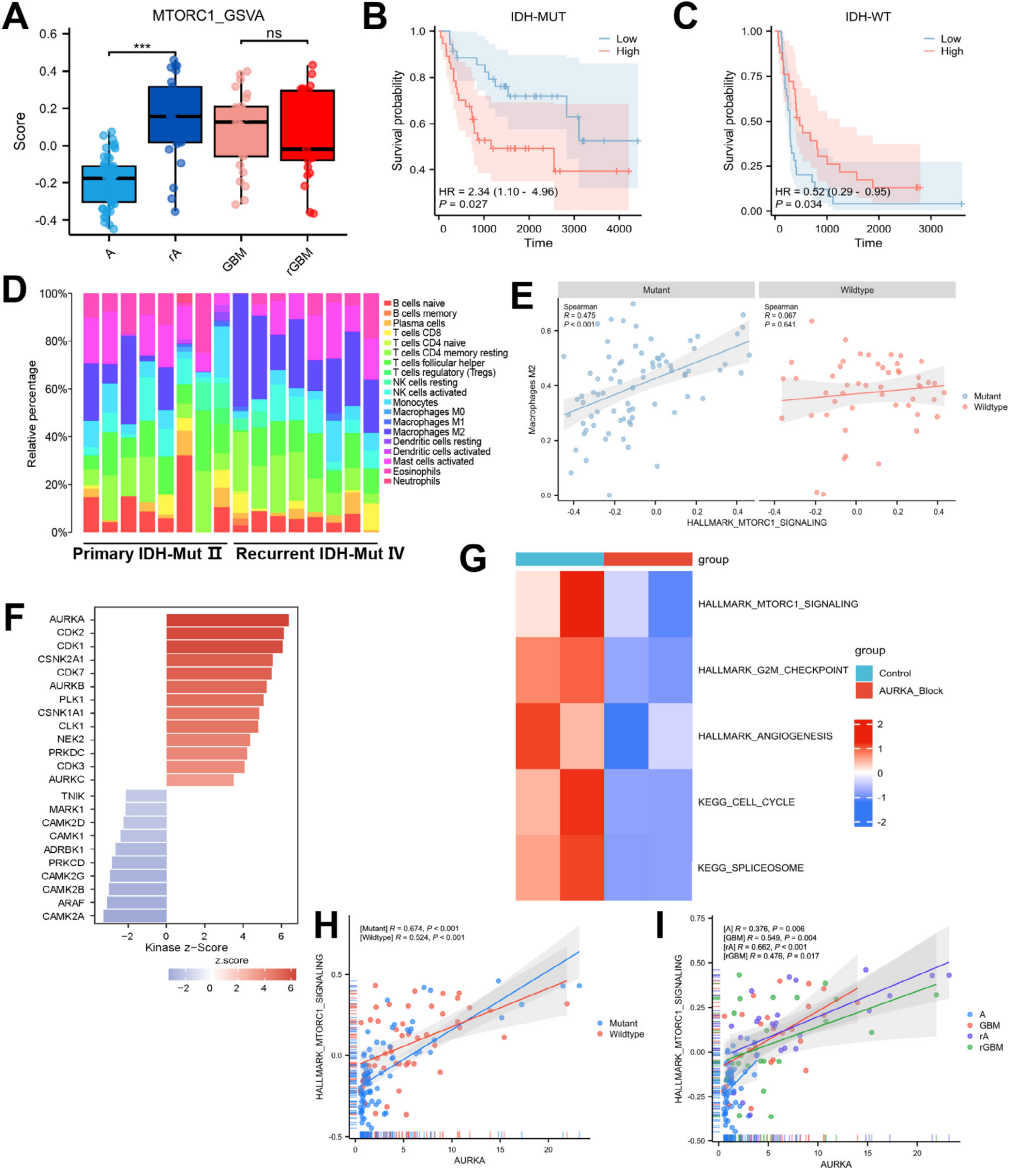
External validation by public datasets. (A) GSVA analysis showing increased mTORC1 activity in recurrent IDH‐mutant gliomas. (B) Survival analysis of mTORC1‐high vs. mTORC1‐low IDH‐mutant gliomas. (C) Survival analysis of mTORC1‐high vs. mTORC1‐low IDH‐wildtype gliomas. (D) Transcriptomic‐based deconvolution analysis. (E) Correlation between mTORC1 and M2 macrophages in gliomas with different IDH statuses. (F) Kinase enrichment analysis from phosphoproteomics and proteomics. (G) GSVA comparison of mTORC1, angiogenesis, and G2M activity after AURKA inhibition. (H) Correlation between mTORC1 and AURKA in gliomas with different IDH statuses. (I) Correlation between mTORC1 and AURKA in primary vs. recurrent IDH‐mutant gliomas. ***p < 0.001.

Data from public databases indicated that there were no significant differences in the mTORC1 pathway changes between IDH wild‐type gliomas before and after recurrence (Figure 2A). Survival analysis suggested that activation of the mTORC1 pathway had a minimal impact on survival time in IDH wild‐type gliomas, while it was significantly associated with poor prognosis in IDH‐mutant gliomas (Figure 2B,C).

Given the activation of certain immune‐related pathways identified in the enrichment analysis, we performed a deconvolution analysis on our RNA‐seq cohort data. The results demonstrated a significant increase in the proportion of M2 macrophages within the recurrent group, which was statistically different from the primary group (Figure 2D). To investigate this phenomenon further, we analyzed the correlation between M2 macrophages and the mTORC1 pathway. In the CGGA database, we found that the correlation between the mTORC1 pathway and M2 macrophages was more pronounced in IDH‐mutant gliomas compared to IDH wild‐type gliomas (Figure 2E).

Additionally, we conducted KSEA on the differentially phosphorylated sites, revealing the enrichment of several kinases during the recurrence of IDH‐mutant gliomas, including aurora kinases (AURKA/AURKB/AURKC), cyclin‐dependent kinases (CDK1/CDK2/CDK7), and casein kinase (CSNK2A1/CSNK1A1) (Figure 2F). These kinases have previously been reported to be associated with gliomas. To further validate these findings, we analyzed a dataset from the GEO database that included samples with AURKA inhibition and their corresponding controls. The analysis indicated that in samples with AURKA inhibition, both the mTORC1 pathway and its related pathways were downregulated (Figure 2G). Moreover, correlation analysis from the CGGA database highlighted a strong association between AURKA and mTORC1 in recurrent IDH‐mutant gliomas (Figure 2H,I). In summary, we propose that AURKA may contribute to the recurrence of IDH‐mutant gliomas through the activation of the mTORC1 pathway.

### 3.3 Macrophage Subpopulation Associated With IDH‐Mut Glioma Progression Exhibiting High mTORC1 Activity and Angiogenesis

Our multi‐omics analysis suggested that mTORC1 may influence the immune microenvironment, thereby affecting the recurrence and progression of IDH‐mutant gliomas. In contrast, mTORC1 appears to have no significant impact on the progression of IDH‐wildtype gliomas. To further investigate the specific role of mTORC1 in IDH‐mutant gliomas, we analyzed single‐cell data from GSE166218. This dataset includes four groups of mouse models: two implanted with IDH‐mutant glioma cells and two with IDH‐wildtype cells, with one group from each mutation type sacrificed at 7 days and the other at 28 days. CD45^+^ cells were isolated for scRNA sequencing. This data provide a dynamic representation of the glioma immune microenvironment under different IDH mutation statuses across various time points.

**FIGURE 3:**
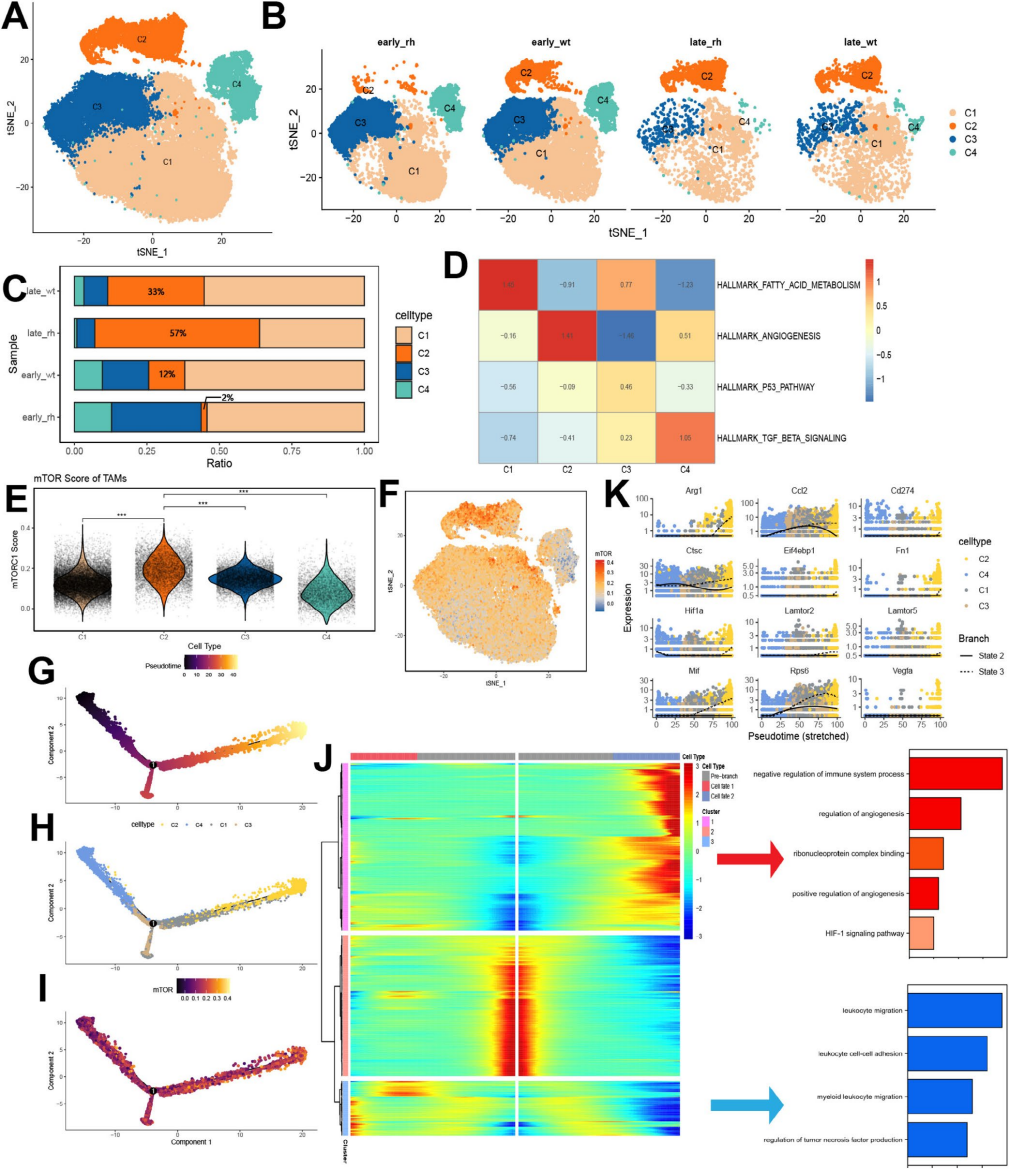
Identification of significantly increased angio‐TAMs in mouse models. (A) t‐SNE plot of macrophages. (B) t‐SNE plot of macrophages at different time points and IDH mutation statuses. (C) Horizontal proportion of macrophages at different time points and IDH mutation statuses. (D) GSVA evaluates the function of different macrophage subpopulations. (E) Violin plot of mTORC1 scores in macrophages. (F) t‐SNE feature plot of mTORC1 scores in macrophages. (G–I) Pseudotime trajectory of macrophages. (J) BEAM algorithm and enrichment analysis in C2 subpopulation development. (K) Expression changes of key M2 polarization and angiogenesis genes along the pseudotime trajectory.   ***p < 0.001.

We first performed AddmodelScore on all cell populations, and the results showed that myeloid‐derived mouse CD45^+^ cells exhibited higher mTORC1 scores (Figure S1G). Therefore, we selected the macrophage cells for further analysis. Dimensionality reduction and clustering identified four distinct macrophage subpopulations (Figure 3A, Figure S1H). Notably, the C2 cluster exhibited the most pronounced differences between IDH‐mutant glioma subgroups at early and late stages, and this cluster also represented the largest proportion of tumor‐associated macrophages in the late_rh group (Figure 3B,C). These findings suggest that the C2 cluster plays a crucial role in the progression of IDH‐mutant gliomas. GSVA revealed that the C2 cluster is associated with angiogenesis functions, with high expression of VEGFA and HIF1A. Furthermore, this subpopulation displayed the highest mTORC1 activity (Figure 3D–F). Pseudotime analysis indicated that the C2 cluster is at the terminal developmental stage of TAMs, and it exhibited the highest mTORC1 scores (Figure 3G–I). Using the BEAM algorithm, we identified that this subcluster is enriched in immune‐suppressive, angiogenesis, and hypoxia‐related pathways, all of which are associated with malignant progression in gliomas. In contrast, the opposing cell subpopulations were enriched for anti‐tumor signaling pathways, such as leukocyte migration and tumor necrosis factor secretion (Figure 3J, Table S5). Trajectory analysis further demonstrated that during the evolution towards the C2 cluster, there was a significant upregulation of mTORC1‐related molecules, M2 polarization‐related genes, and angiogenesis‐related gene sets. These findings suggest that the C2 cluster may play a critical role in the progression of IDH‐mutant gliomas (Figure 3K).

### 3.4 Single‐Cell Sequencing Reveals mTORC1‐Driven Dominance of MoTAMs and MES Subpopulation in the Recurrence and Progression of IDH‐Mutant Gliomas

**FIGURE 4:**
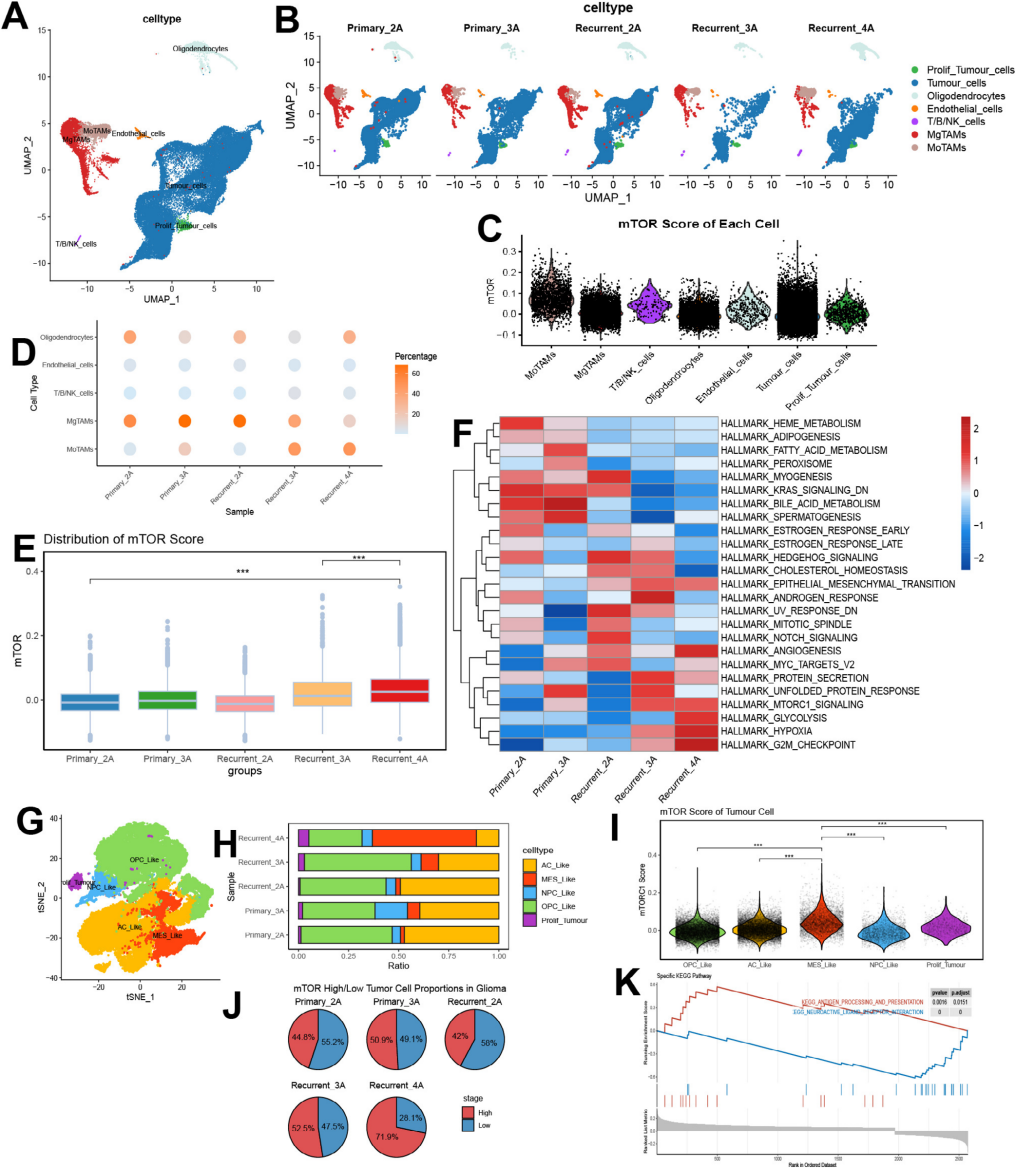
Omics landscape of primary and recurrent IDH‐mutant gliomas in humans. (A) UMAP plot of IDH‐mutant gliomas. (B) UMAP subplots of IDH‐mutant gliomas at different grades and states. (C) Violin plot of mTORC1 scores across different cell subpopulations. (D) Horizontal proportions of non‐tumor cell subpopulations in gliomas at different grades and states. (E) Boxplot of mTORC1 scores in different grades and states. (F) Heatmap of GSVA scores based on the HALLMARKER for IDH‐mutant gliomas at grades and states. (G) t‐SNE plot of tumor cell. (H) Horizontal proportions of tumor cell subpopulations in gliomas at different grades and states. (I) Violin plot of mTORC1 scores across different tumor cell subpopulations. (J) Horizontal proportions of mTORC1 high and low subgroups. (K) GSEA analysis based on KEGG pathways between mTORC1 high and low subgroups. ***p < 0.001.

Given that we identified a group of mTORC1‐active, angiogenesis‐related tumor‐associated macrophages (Angio‐TAMs) in the mouse single‐cell data, which are closely associated with IDH mutation, we need to further explore the interactions of this subpopulation with other tumor components and its role in the recurrence and progression of IDH‐mutant gliomas. To investigate this, we conducted single‐cell analyses using matched samples of IDH‐mutant primary and recurrent gliomas from public databases. In this process, we extracted a total of 48,655 cells and through dimensionality reduction and cell annotation, we initially categorized the cells into seven subpopulations: tumor cells, proliferative tumor cells, oligodendrocytes, endothelial cells, T/B/NK cells, monocyte‐derived tumor‐associated macrophages (MoTAMs), and microglia‐derived tumor‐associated macrophages (MgTAMs) (Figure 4A, Figure S2A).

To investigate the activation levels of mTORC1 across different cell subpopulations, we employed the AddmodelScore method to score each subpopulation. The results indicated that MoTAMs exhibited significantly higher mTORC1 scores compared to other subpopulations, with this population being particularly pronounced in recurrent and high‐grade gliomas (Figure 4B–D). Furthermore, when we compared overall single‐cell mTORC1 scores, the results aligned with those from bulk tissue‐level analyses, further supporting this finding (Figure 4E). Finally, we performed GSVA scoring on the single‐cell transcriptomic data based on Hallmark gene sets. The results showed a significant increase in mTORC1 scores in recurrent high‐grade gliomas (Figure 4F). This finding suggests that mTORC1 may play a critical role in the recurrence and progression of gliomas, particularly within MoTAMs, indicating that this population may be the primary cellular source of mTORC1 activation in recurrent IDH‐mutant gliomas.

To further understand the broader role of mTORC1 in glioma progression, we next examined the mTORC1 activity in tumor cells. After performing further dimensionality reduction and clustering, we conducted GSVA scoring based on previously published molecular subtype markers for gliomas, successfully annotating five subpopulations: AC, MES, OPC, NPC, and proliferative tumor cells (Figure 4G, Figure S2B, Table S6). Notably, the MES subpopulation showed a significant increase in proportion within grade IV recurrent gliomas, corroborating earlier findings (Figure 4H, Figure S2C). Using the AddmodelScore assessments, we observed that the mTORC1 scores in the MES subpopulation were markedly higher than those in other subpopulations (Figure 4I). To further analyze the role of mTORC1 in different glioma samples, we stratified the samples into high and low groups based on the median mTORC1 score. The results indicated a significantly increased proportion of the mTORC1 high group among grade IV recurrent gliomas (Figure 4J). Additionally, we performed GSEA on the differentially expressed genes between the high and low mTORC1 groups. The analysis revealed that the high mTORC1 activity group was predominantly enriched in antigen presentation pathways, which are consistent with our bulk‐level analysis (Figure 4K). These findings suggest that the activation of the mTORC1 signaling pathway is closely associated with glioma recurrence and malignant progression, particularly within the MES subpopulation. In summary, the mTORC1 signaling pathway is significantly upregulated in the MES‐like glioma cell subpopulation, which represents the highest proportion in IDH‐mutant recurrent gliomas and is closely linked to the level of recurrence.

### 3.5 mTORC1‐Activated Angio‐TAM Subpopulation Drives Recurrence and Progression of IDH‐Mutant Gliomas via M2 Polarization and Angiogenesis

**FIGURE 5:**
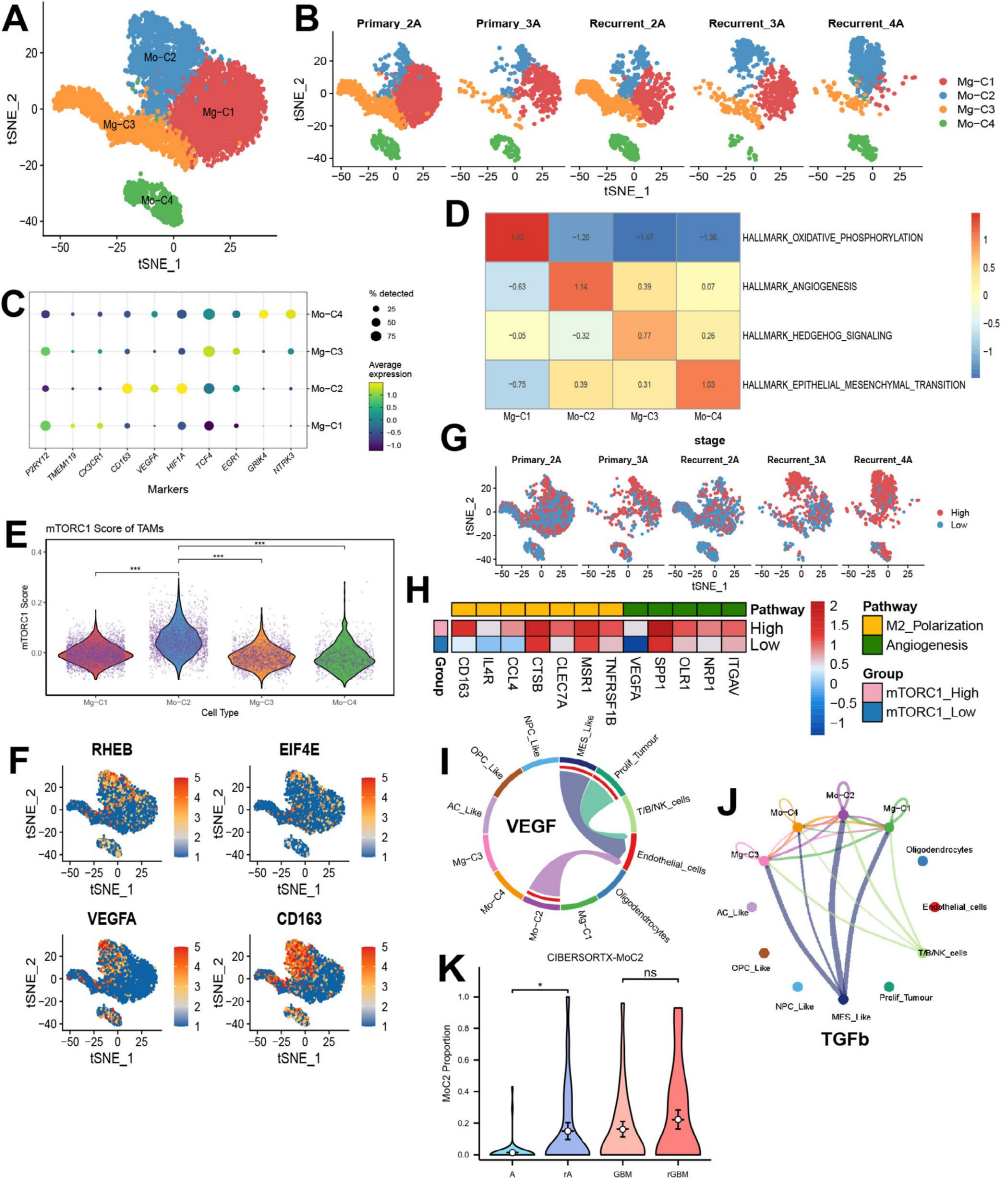
Landscape of TAMs in primary and recurrent IDH‐mutant gliomas. (A) t‐SNE plot of TAMs. (B) t‐SNE subplots of TAMs at different grades and states of IDH‐mutant gliomas. (C) Cluster annotation dot plot of TAMs. (D) GSVA analysis evaluates the functional states of different TAMs subpopulations. (E) Violin plot of mTORC1 scores across different TAMs subpopulations. (F) t‐SNE feature plot in TAMs. (G) t‐SNE plot of TAMs grouped by high and low mTORC1 scores. (H) Heatmap of M2 polarization and angiogenesis molecules in high and low mTORC1 groups. (I) Cellchat landscape of VEGF signaling in IDH‐mutant gliomas. (J) Cellchat landscape of TGF‐β signaling in IDH‐mutant gliomas. (K) Deconvolution analysis of the C2 subpopulation based on the CGGA database. *p < 0.05, ***p < 0.001.

The role of the immune microenvironment, particularly TAMs, is gaining increasing attention in glioma research. To validate that the cell subset C2 identified in our IDH‐mutant mouse model exhibits similar functional characteristics in human glioma tissues, we performed an in‐depth analysis of macrophage subpopulations, categorizing them into four groups, with MgTAMs and MoTAMs further divided into two subgroups each. Our findings indicate that MgTAMs are predominantly distributed in low‐grade and primary glioma subtypes. The Mo‐C2 group, characterized by markers such as VEGFA and HIF1A, dominates in high‐grade and recurrent gliomas, also expressing M2‐related markers like CD163 (Figure 5A–C, Figure S2D,E). We propose that the Mo‐C2 subset represents a distinct population of Angio‐TAMs (Figure 5D). Using AddModelScore analysis, we found that subpopulations with high mTORC1 scores were predominantly concentrated in the Mo‐C2 group, a finding further supported by violin plot analysis (Figure 5E). This aligns with the functional characteristics of the distinct macrophage population we identified in the mouse model. Using the median mTORC1 score, we classified macrophages into high and low mTORC1 groups. We found that the high mTORC1 group was predominantly enriched in recurrent grade IV gliomas (Figure 5G). Moreover, the high mTORC1 group exhibited elevated expression of several genes associated with mTORC1 signaling, M2 polarization, and angiogenesis, including RHEB, EIF4E, CD163, and VEGFA, compared to the low mTORC1 group (Figure 5F,H).

To investigate the potential signaling functions of this subgroup, we conducted cell communication analysis, revealing a significant cross‐talk between the VEGF pathway and MES cells with this macrophage subgroup and vascular endothelial cells. This suggests that mTORC1 signaling activation may enhance tumor angiogenesis by regulating VEGF expression, contributing to glioma progression and poor prognosis (Figure 5I, Figure S2F). Both the MES subset and the C2 subset showed the most significant cross‐talk with the TGF‐β signaling pathway, suggesting that the MES subset may educate macrophages through this pathway (Figure 5J, Figure S2G).

To further investigate the impact of this macrophage subpopulation on glioma patients, we employed CIBERSORTX to deconvolute this subpopulation within the bulk data from the CGGA. Comparative analyses revealed that the proportion of this subpopulation showed little variation between primary and recurrent IDH wild‐type gliomas. However, in IDH‐mutant gliomas, the proportion of this subpopulation was significantly elevated in recurrent tumors compared to primary gliomas, suggesting a critical role for this subpopulation in the recurrence progression of gliomas (Figure 5K).

### 3.6 Spatial Transcriptomics Reveals mTORC1‐Activated Angio‐TAMs and Their Interactions With MES and Endothelial Cells in IDH‐Mutant Gliomas

**FIGURE 6:**
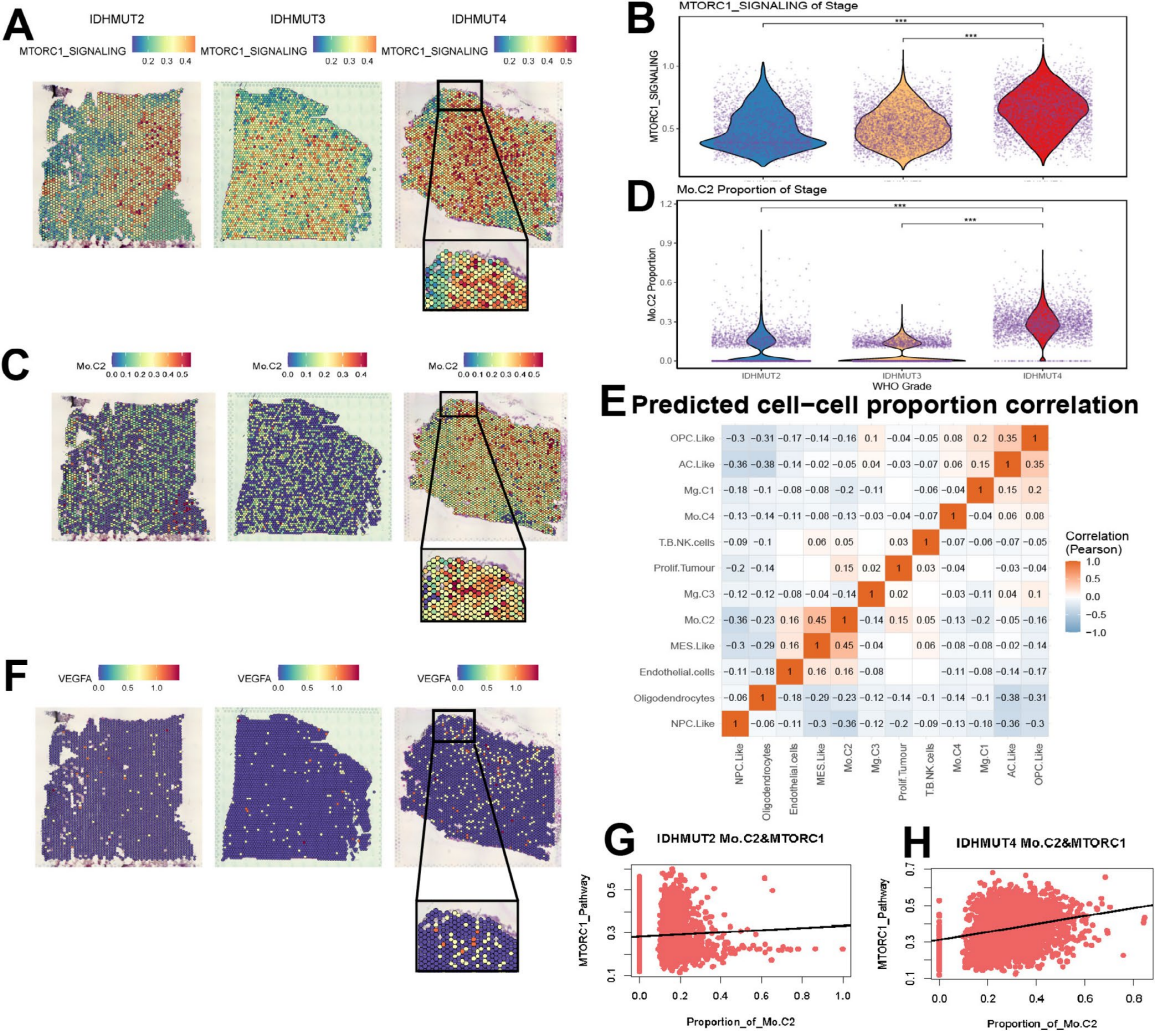
Spatial Transcriptomics Reveals Co‐Localization of mTORC1, C2 Subpopulation, and Angiogenesis in IDH‐Mutant Gliomas. (A) GSVA feature plot of mTORC1 in different grades of IDH‐mutant gliomas. (B) Violin plot of mTORC1 in different grades of IDH‐mutant gliomas. (C) SPOTLIGHT deconvolution feature plot of the C2 subpopulation in spatial transcriptomics. (D) Violin plot of C2 subpopulation activity in spatial transcriptomics. (E) Spatial cell–cell communication analysis plot. (F) Feature plot of VEGFA expression in spatial transcriptomics. (G, H) Correlation analysis of mTORC1 scores and C2 subpopulation in spatial transcriptomics. ***p < 0.001.

To address the limitations of single‐cell analysis in depicting spatial relationships within gliomas, we utilized spatial transcriptomics data from different grades of IDH‐mutant gliomas to conduct an in‐depth analysis of relevant signaling pathways and cell population distributions. Our results indicate that mTORC1 activation levels are significantly elevated in high‐grade gliomas (Figure 6A,B). Through deconvolution analysis, we found that the spatial positioning of the Mo‐C2 group, MES cells, and vascular endothelial cells aligned with conclusions drawn from single‐cell data (Figure 6C,D, Figure S2H,I). Further spatial cell communication analysis revealed a substantial intensity of interactions among the Mo‐C2 group, MES cells, and endothelial cells, highlighting their interactivity within the glioma microenvironment (Figure 6E). The key angiogenic signaling molecule VEGFA exhibits spatial co‐localization with both mTORC1 and the Mo‐C2 TAM subset (Figure 6F). Notably, our correlation analysis demonstrated that macrophages exhibited the highest spatial correlation with mTORC1 in high‐grade gliomas (Figure 6G,H). These findings provide critical insights into cellular interactions within the glioma microenvironment and their implications for tumor progression, particularly in the context of activated mTORC1 signaling pathways.

## 4 Discussion

As research on gliomas continues to advance, our understanding of this disease has expanded from transcriptomics to proteomics and phosphoproteomics. This transition aids in a deeper comprehension of the pathogenesis of gliomas. Previous studies have shown that differential analyses of transcriptomes and proteomes often reveal inconsistencies between RNA and protein expression in various tumors [32, 33]. To address this issue, we performed differential analyses on transcriptomic, proteomic, and phosphoproteomic data and conducted enrichment analyses on the differentially expressed genes and proteins. Our results indicated that pathways related to spliceosomes, the cell cycle, and DNA repair were consistently upregulated across transcriptomic, proteomic, and phosphoproteomic datasets. This suggests that these pathways may reflect changes in protein and phosphorylation levels as indicated by transcriptomic data. Furthermore, GSEA revealed that the mTORC1, cell cycle, and angiogenesis pathways were significantly upregulated at the transcript level in the recurrence progression group. Significant phosphorylation changes occurred at key sites during tumor recurrence, particularly on EIF4EBP1 (Thr70 and Thr46) and STAT3 (Tyr705). These phosphorylation events were associated with mTORC1 activation in recurrent gliomas with IDH mutations. Phosphorylation of EIF4EBP1 releases its inhibition of EIF4E, promoting tumor proliferation and migration. Similarly, phosphorylation of STAT3 contributes to angiogenesis and tumor growth [34, 35]. Utilizing GSVA scoring, we found that these pathways also exhibited consistency in the proteomic results, aligning with previous findings [6, 36]. Given the relatively small sample size of our study, we further validated and explored our findings using the CGGA database. The results indicated that compared to IDH wild‐type gliomas, IDH‐mutant gliomas exhibited significant upregulation of the mTORC1 pathway during recurrence progression, along with notable differences in survival time. These findings provide important evidence for our understanding of the biological characteristics of gliomas.

Previous studies have shown that the unique immune microenvironment of gliomas significantly influences their malignant invasion and recurrence, with the proportion of M2 macrophages identified as a reliable indicator of glioma prognosis [37, 38, 39]. We further analyzed the changes in the proportion of M2 macrophages in our recurrent glioma samples. The results indicated that the proportion of M2 macrophages significantly increased in IDH‐mutant gliomas after recurrence progression, suggesting a potential association with poor prognosis. To verify whether the increase in M2 macrophages was related to the activation of the mTORC1 pathway, we conducted a correlation analysis using the CGGA database. The results showed a stronger correlation between mTORC1 and the proportion of M2 macrophages in IDH‐mutant gliomas, suggesting that the mTORC1 pathway may partially participate in the remodeling of the immune microenvironment in IDH‐mutant gliomas. Further KSEA enrichment analysis indicated that AURKA might be a key kinase activated during the recurrence progression of IDH‐mutant gliomas. Through public databases, we utilized GSVA to explore downregulated pathways in glioma samples with AURKA inhibition, revealing significant downregulation of pathways such as mTORC1, the cell cycle, and angiogenesis after AURKA suppression. Additionally, the CGGA database demonstrated a more significant correlation at the transcript level between AURKA and recurrent IDH‐mutant gliomas. AURKA has been implicated in regulating the cell cycle through the activation of the PI3K/AKT/mTOR pathway, and dysregulation of AURKA in gliomas has been shown to promote tumorigenesis [40, 41]. Previous studies have demonstrated that AURKA can promote tumor progression in lung cancer by nuclear translocation, which relieves the inhibition of mTORC1 signaling. Moreover, in preclinical breast cancer models, the combination of AURKA inhibitor alisertib with mTORC1 inhibitor TAK‐228 enhanced antitumor activity, increasing cell death, and apoptosis [42, 43].

Our results suggest that the inhibition of AURKA in gliomas may suppress the mTORC1 pathway and inhibit tumor angiogenesis. These results suggest that the phosphorylation induced by AURKA activation may be one of the critical initiating points for the recurrence progression of IDH‐mutant gliomas.

Based on this, we hypothesize that IDH‐mutant gliomas may exhibit partial polarization of TAMs under the influence of mTORC1, leading to the conversion of this subset towards a pro‐tumorigenic phenotype. This phenomenon appears to be less pronounced in IDH wild‐type gliomas. To test this hypothesis, we analyzed single‐cell sequencing data from CD45^+^ cells in GL261 xenografted mice with both IDH wild‐type and mutant backgrounds using public databases. After dimensionality reduction, clustering, and subgroup extraction, we identified that the C2 group of TAMs exhibited high mTORC1 activity, with characteristics such as active angiogenesis and M2 polarization. This subgroup was most prominent in IDH‐mutant mice 28 days post‐tumor implantation, with a significant difference in the proportions of early and late‐stage cells compared to wild‐type mice. These findings suggest a more critical role for this subgroup in the progression of IDH‐mutant gliomas. These results lead us to hypothesize that during the progression of IDH‐mutant gliomas, activation of the mTORC1 pathway may trigger specific Angio‐TAMs with angiogenesis properties, which subsequently play a tumor‐promoting role. Angio‐TAMs have been identified in various tumors and are characterized by the high expression of marker genes such as SPP1 and FN1 [44, 45, 46]. FN1, in particular, has been recognized for its role in promoting tumor recurrence, although the specific signaling pathways involved remain unclear [47]. We propose that the activation of the mTORC1 pathway could be one of the key biological mechanisms driving the dominance of this macrophage subset during the recurrence and progression of IDH‐mutant gliomas.

Previous studies have identified TAM subpopulations closely associated with the IDH mutation status in mouse glioma models. Given the inherent heterogeneity between humans and mice, we further investigated whether this specific TAM subgroup is also present in human IDH‐mutant glioma cases during glioma recurrence and progression and explored its role within the immune microenvironment. Our results showed that mTORC1 activity was significantly higher in the recurrent high‐grade gliomas group, consistent with our previous observations. Notably, MoTAMs exhibited the highest mTORC1 activity, suggesting that mTORC1 may promote glioma recurrence by influencing MoTAMs within the immune microenvironment. Additionally, further analysis of tumor subpopulations revealed that the MES subtype had the highest mTORC1 scores. This subtype was also enriched for signaling pathways associated with antigen presentation and other immune‐related pathways, indicating a potential link to tumor immune microenvironment remodeling [48, 49]. These findings underscore the potential mechanisms by which mTORC1 regulates both the tumor microenvironment and tumor cell behavior.

Recent studies have underscored the functional diversity of TAMs by employing a more refined annotation method that goes beyond the traditional M1 and M2 classifications, revealing the complexity of the immune microenvironment [39]. In our study, we further subdivided TAMs in gliomas into four distinct categories with the MoTAM subtype being divided into Mo‐C2 and Mo‐C4. We successfully isolated this angiogenesis‐associated macrophage subset from human glioma samples using single‐cell sequencing techniques. The Mo‐C2 subtype was found to strongly express pathways associated with tumor angiogenesis, such as CD163 and VEGFA. Notably, a consistent correlation was observed between Mo‐C2 and mTORC1 activity, with the highest proportion of this subtype detected in the recurrence group of high‐grade gliomas, which also exhibited the highest mTORC1 scores. We have reason to believe that this subgroup aligns with the Angio‐TAMs we identified in the mouse model, characterized by highly active mTORC1 signaling, elevated levels of angiogenesis, and M2 polarization. Therefore, we consider this a distinct, angiogenesis‐associated macrophage population. Further analysis of cell communication confirmed that this Angio‐TAM subtype interacts with endothelial cells through the VEGF signaling pathway, suggesting that Angio‐TAMs may promote angiogenesis via the mTORC1 pathway, thereby contributing to glioma recurrence and progression. This phenomenon is also similar to the hypoxia‐vascular niche observed in glioblastoma, which promotes glioma progression [49, 50, 51]. Additionally, this subgroup showed the strongest correlation with the TGF‐β signaling pathway in MES cells, suggesting a potential link between MES‐induced macrophage polarization and tumor progression. Moreover, we employed spatial transcriptomics to investigate the spatial distribution of mTORC1‐active regions and the Angio‐TAMs subgroup, discovering a significant co‐localization phenomenon in high‐grade IDH‐mutant glioma tissues. Spatial cell communication analysis further validated our previous findings. These discoveries provide new insights into the functions of TAMs within the glioma microenvironment and their roles in tumor progression.

Although multiple datasets were utilized in this study, the sample size was relatively small, comprising only 16 samples from 8 patients. To strengthen the robustness and generalizability of our findings, we plan to expand the cohort in future studies, including a larger number of patients and samples from various clinical stages of glioma. Moreover, we will focus on investigating how the AURKA/mTORC1 axis mediates macrophage polarization towards an angiogenesis‐related phenotype and further explore potential therapeutic targets. These efforts will provide a more comprehensive understanding of the mechanisms.

In conclusion, our study highlights the potential involvement of the AURKA‐mTORC1 pathway in the recurrence progression of IDH‐mutant gliomas, a process partially mediated by the induction of VEGFA expression in TAMs, which in turn promotes angiogenesis. Additionally, we identified a distinct population of Angio‐TAMs in the recurrent IDH‐mutant glioma tissues. This subgroup is characterized by active mTORC1 signaling, M2 polarization, and enhanced angiogenesis, suggesting a close association with the recurrence and progression of IDH‐mutant gliomas. Thus, deepening our understanding of the role of mTORC1 may significantly advance our knowledge of the mechanisms underlying glioma recurrence and provide valuable insights for the development of targeted therapeutic strategies for these tumors.

## Author Contributions

Y.L., S.L., and M.L. designed the experiment, interpreted the data, and wrote the manuscript. X.W. collected and processed the clinical samples. J.G., Y.K., and H.T. performed bioinformatic analysis and interpreted the results. Y.B. and L.G. contributed to drafting and revising the manuscript. Q.S. and J.Y. contributed to analysis of clinical data and revised the manuscript. X.W., J.G., and H.T. contributed equally to this manuscript. All authors read and approved the final manuscript.

## Ethics Statement

The studies involving human participants were reviewed and approved by the Ethical Committee for Human Investigation of the Shanghai General Hospital (2021SQ054). The patients provided their written informed consent to participate in this study.

## Conflicts of Interest

The authors declare no conflicts of interest.

## Supporting information


**Appendix S1** Supplemental experimental procedures.

**Figure S1** Multi‐omics landscape of primary and recurrent IDH‐mutant gliomas and differential activation of mTORC1 pathway at single‐cell resolution(A) Volcano plot of differentially expressed genes. (B) Volcano plot of differentially expressed proteins. (C) Volcano plot of differentially phosphoproteomic sites. (D) Fold changes and correlation of proteins and phosphorylated sites between recurrent and primary groups. (E) Heatmap of mTORC1 pathway expression at the transcriptional level. (F) Heatmap of mTORC1 pathway at the phosphoproteomic level. (G) Violin plots of AddModuleScore for the mTORC1 pathway across subclusters. (H) Dot plot annotation of macrophage subcluster markers in mouse IDH‐mutant gliomas.

**Figure S2** Heterogeneous cellular proportion, functional distribution, and potential intercellular crosstalk in IDH‐mutant gliomas of different grades (A) Marker annotation of single‐cell subclusters in IDH‐mutant gliomas. (B) GSVA scores for tumor cell subclusters. (C) t‐SNE distribution of tumor cell subclusters across different glioma grades. (D) Dot plot annotation of macrophage subcluster markers in IDH‐mutant gliomas. (E) Bar plot showing proportions of macrophage subclusters in IDH‐mutant gliomas. (F) Violin plots of key crosstalk markers in the VEGF pathway. (G) Dot plot of key crosstalk markers in the TGF‐β pathway. (H‐I) SPOTLIGHT deconvolution feature plot of the MES‐like and endothelial cells in spatial transcriptomics.

**Table S1** Distribution matrix of transcriptomic differential analysis results
**Table S2** Distribution matrix of proteomic differential analysis results.
**Table S3** Distribution matrix of phosphoproteomic differential analysis results.
**Table S4** Hallmark mTORC1 gene set.
**Table S5** Top 1000 gene sets screened by the BEAM algorithm.
**Table S6** Characteristic markers of tumor cell subclusters.

## Data Availability

The data that support the findings of this study are available from the corresponding author upon reasonable request.
