# Supplementary material for: Single‐Cell Profiling and Proteomics‐Based Insights Into mTORC1‐Mediated Angio+TAMs Polarization in Recurrent IDH‐Mutant Gliomas
Source: CNS Neurosci Ther. 2025 Apr 9;31(4):e70371. doi: 10.1111/cns.70371 (PMC11979715; doi:10.1111/cns.70371)
Supplement: Supplementary file 1 — Appendix S1 Supplemental experimental procedures. [file CNS-31-e70371-s001.docx]

**Supplemental Experimental Procedures**

**RNA extraction**

RNA was extracted from tissues by using the RNA storm FFPE kit (CELLDATA, USA, #CA94538) according to the manufacturer’s protocol. RNA integrity and concentration were determined using a Nano Drop 8000 spectrophotometer (Thermo Fisher Scientific). For library preparation of RNA sequencing, a total amount of 500 ng RNA per sample was used as input material for RNA sample preparations. Sequencing libraries were generated using a Ribo-off rRNA Depletion Kit (H/M/R) (Vazyme, Nanjing, China, #N406) and a VAHTS Universal V6 RNA-seq Library Prep Kit for Illumina (#N401-NR604) following the manufacturer’s recommendations. Index codes were added to attribute sequences to each sample. The libraries were sequenced on an Illumina platform and 150 bp paired-end reads were generated.

**mRNA-Sequencing Library establishment and quality control**

0.1-1 μg of total RNA per sample was prepared for library preparation. The NEB Next® Poly(A) mRNA Magnetic Isolation Module and NEB Next® Ultra™ II mRNA Library Prep Kit for Illumina® were used for mRNA isolation and library construction following the manufacturers’ protocols. As for the quality control of library, Qubit dsDNA HS Assay Kit was used to measure the concentration of library, then Agilent 4200 was used to examine the distribution of segments in library. Finally, library molar concentration was determined using the KAPA Library Quant kit (illumina) universal qPCR Mix. High-throughput transcriptome sequencing was performed on an Illumina NovaSeq 6000 platform according to the manufacturer’ s instructions.
**Proteome and Phosphoproteome Sample Preparation**

The sample preparation followed FFomic strategy. Accurate evaluation of tumor cellularity was determined using the middle section of each tumor tissue block, which was resected and subjected to hematoxylin and eosin (H&E) staining. For proteomic, phosphoproteomic sample preparation, slides (10 μm thick) were sectioned, deparaffinized with xylene, and washed in an ethanol gradient. Specimens selected according to H&E staining were scraped using a dissecting microscope and then stored at − 80 °C until needed.

**FFPE protein extraction and trypsin digestion :**

Samples were lysed in TCEP buffer (2% deoxycholic acid sodium salt, 40 mM 2-chloroacetamide, 100 mM Tris–HCl, 10 mM Tris(2-chloroethyl) phosphate, 1 mM PFSM, pH 8.5) supplemented with protease inhibitors and phosphatase at 99 °C for 30 min. After cooling to room temperature, trypsin was added and digested for 18 h at 37 °C. 10% formic acid was added and vortexed for 3 min, followed by sedimentation for 5 min (12,000g). Next, a new 1.5-mL tube with extraction buffer (0.1% formic acid in 50% acetonitrile) was used to extract the supernatant (vortex for 3 min, followed by 12,000g of sedimentation for 5 min). Collected supernatant was transferred into a new tube for drying using a SpeedVac.

**Phospho-peptide enrichment :**

Tryptic peptides were used for phosphopeptide enrichment using a High- Select Fe-NTA kit (Thermo Fisher Scientific, Rockford, IL, USA, #A32992) according to the kit manual and a previous report [79] with some modifications. In brief, peptides were suspended in binding/wash buffer (contained in the enrichment kit) and mixed with the equilibrated resins. The peptide–resin mixture was incubated for 30 min with three gentle blows at room temperature. Following incubation, the resins were washed thrice with binding/wash buffer and twice with water. The enriched peptides were eluted with elution buffer (contained in the enrichment kit) and immediately dried using a SpeedVac at 30 °C for mass spectrometry analysis.

**Nano-LC–MS/MS analysis**

For the proteome profiling samples, peptides were analyzed on a Q Exactive HF-X Hybrid Quadrupole-Orbitrap Mass Spectrometer (Thermo Fisher Scientific) coupled with a high-performance liquid chromatography system (EASY nLC 1200, Thermo Fisher Scientific). Dried peptide samples re-dissolved in Solvent A (0.1% formic acid in water) were loaded onto a 2-cm self-packed trap column (100 μm inner diameter, 3 μm ReproSil-Pur C18-AQ beads, Dr. Maisch GmbH) using Solvent A and separated on a 150-μm-inner-diameter column with a length of 30 cm (1.9 μm ReproSil-Pur C18-AQ beads, Dr. Maisch GmbH) over a 150-min gradient (Solvent A: 0.1% formic acid in water; Solvent B: 0.1% formic acid in 80% ACN) at a constant flow rate of 600 nL/min (0–150 min, 0 min, 4% B; 0–10 min, 4–15% B; 10–125 min, 15–30% B; 125–140 min, 30–50% B; 140–141 min, 50–100% B; 141–150 min, 100% B). Eluted peptides were ionized at 2 kV and introduced into the mass spectrometer. Mass spectrometry was performed in data-dependent acquisition mode. For the MS1 Spectra full scan, ions with m/z ranging from 300 to 1400 were acquired by an Orbitrap mass analyzer at a high resolution of 120,000. The automatic gain control (AGC) target value was set to 3E+06. The maximal ion injection time was 80 ms. The top 60 precursor ions were selected for fragmentation in an HCD cell with a normalized collision energy of 27%. The resulting fragment ions were transferred to the Orbitrap analyzer, which operated at a resolution of 7500. The automatic gain control (AGC) was set to 5e4 for MS/MS. The maximum ion injection times were set to 20 ms. Dynamic exclusion of previously acquired precursor ions was enabled for 25 seconds.

For the phosphoproteome profiling samples, peptides were analyzed on a OE480 Hybrid Quadrupole-Orbitrap Mass Spectrometer (Thermo Fisher Scientific) coupled with a high-performance liquid chromatography system (EASY nLC 1200, Thermo Fisher Scientific). Dried peptide samples re-dissolved in Solvent A (0.1 % formic acid in water) were loaded onto a 2-cm self-packed trap column (100 μm inner diameter, Dr. Maisch GmbH) using Solvent A and separated on a 150-μm-inner-diameter column with a length of 15 cm (1.9 μm ReproSil-Pur C18-AQ beads, Dr. Maisch GmbH) over a 120-min gradient (Solvent A: 0.1% formic acid in water; Solvent B: 0.1% formic acid in 80% ACN) at a constant flow rate of 600 nL/min (0–120 min, 0 min, 4% B; 0-8 min, 4–15% B; 8–100 min, 15–35% B; 100–110 min, 30–50% B; 110–111 min, 50–100% B; 111–120 min, 100% B). Eluted peptides were ionized at 2.4 kV and introduced into the mass spectrometer. Mass spectrometry was performed in data-dependent acquisition mode. For the MS1 Spectra full scan, ions with m/z ranging from 300 to 1,400 were acquired by an Orbitrap mass analyzer at a high resolution of 120,000. The automatic gain control (AGC) target value was set to 3E+06. The maximal ion injection time was 80 ms. MS2 spectral acquisition was performed in a rapid speed mode with 1s cycletime. Precursor ions were selected and fragmented with higher energy collision dissociation (HCD) with a normalized collision energy of 30 %. Fragment ions were analyzed by an Orbitrap mass analyzer at the resolution of 7,500, with an AGC target at 5E+04. The maximal ion injection time of MS2 was 22 ms. Peptides that triggered MS/MS scans were dynamically excluded from further MS/MS scans for 40 s. The coefficient of variation (CV) values on FAIMS were -45V and -65V.

**Data analysis**

The original data of mass spectrometry analysis were RAW files, and iProteome (https://www.iproteome.com/) one-stop data analysis cloud platform was used for qualitative and quantitative analysis.

**Missing Value Imputation**

For the proteomic and phosphoproteomic data, FOTs multiplied by 1E5 were used for quantification, and missing values were imputed with 1E-5 and finally, log2 transformed, if necessary.
