# Supplementary figures and images for: Single‐Cell Profiling and Proteomics‐Based Insights Into mTORC1‐Mediated Angio+TAMs Polarization in Recurrent IDH‐Mutant Gliomas
Source: CNS Neurosci Ther. 2025 Apr 9;31(4):e70371. doi: 10.1111/cns.70371 (PMC11979715; doi:10.1111/cns.70371)

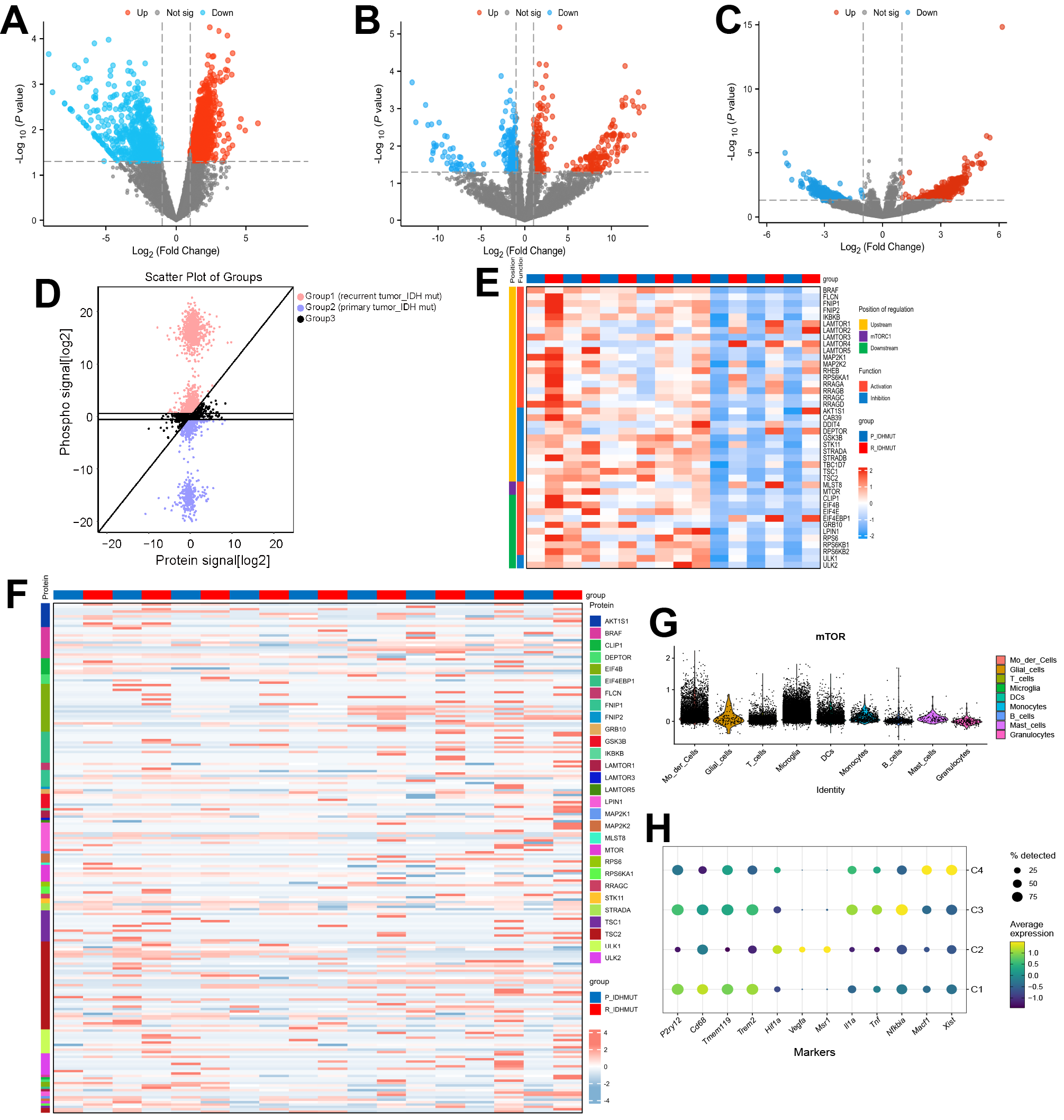

Supplement: Supplementary file 2 — Figure S1 Multi‐omics landscape of primary and recurrent IDH‐mutant gliomas and differential activation of mTORC1 pathway at single‐cell resolution(A) Volcano plot of differentially expressed genes. (B) Volcano plot of differentially expressed proteins. (C) Volcano plot of differentially phosphoproteomic sites. (D) Fold changes and correlation of proteins and phosphorylated sites between recurrent and primary groups. (E) Heatmap of mTORC1 pathway expression at the transcriptional level. (F) Heatmap of mTORC1 pathway at the phosphoproteomic level. (G) Violin plots of AddModuleScore for the mTORC1 pathway across subclusters. (H) Dot plot annotation of macrophage subcluster markers in mouse IDH‐mutant gliomas. [file CNS-31-e70371-s003.tif]

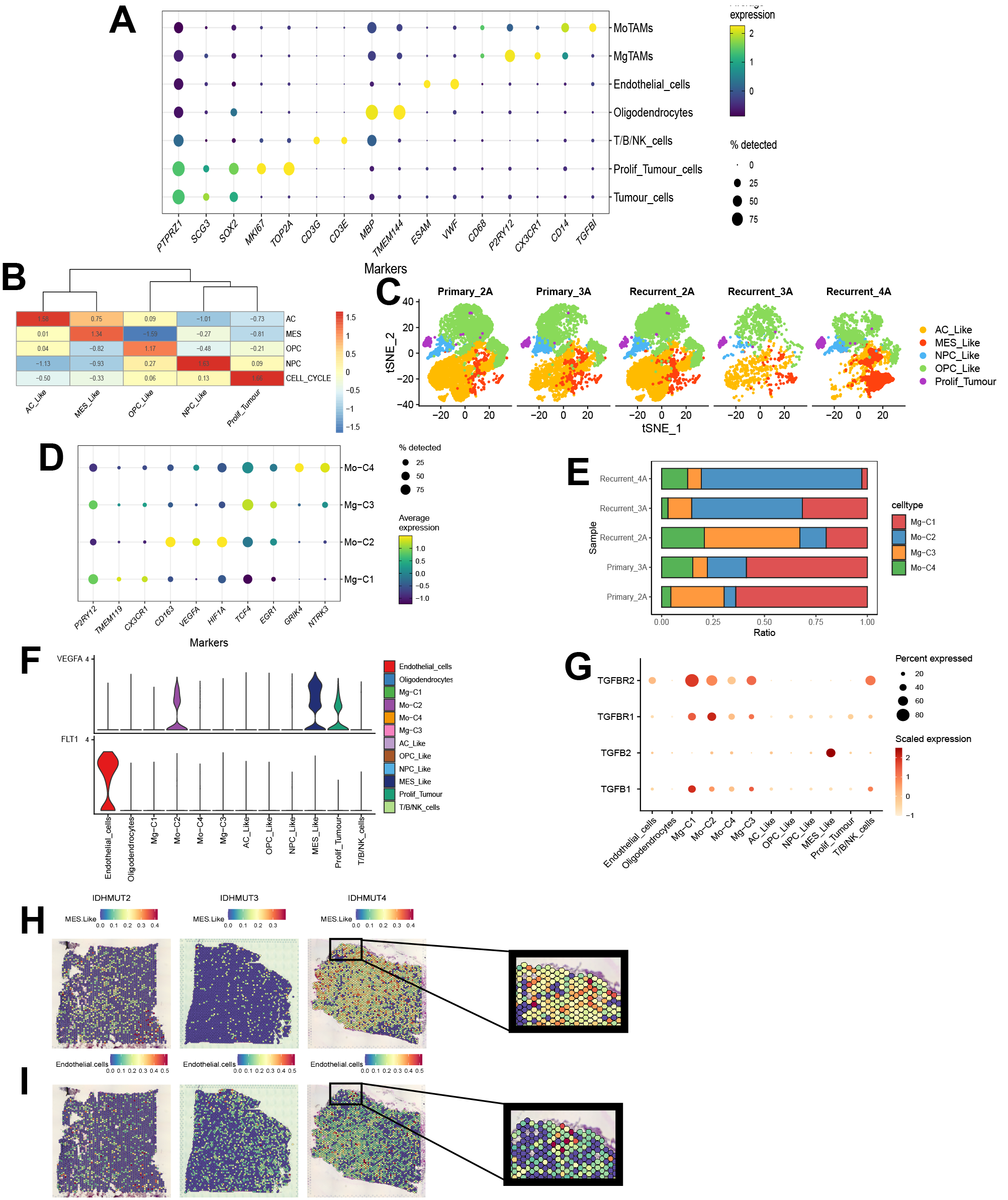

Supplement: Supplementary file 3 — Figure S2 Heterogeneous cellular proportion, functional distribution, and potential intercellular crosstalk in IDH‐mutant gliomas of different grades (A) Marker annotation of single‐cell subclusters in IDH‐mutant gliomas. (B) GSVA scores for tumor cell subclusters. (C) t‐SNE distribution of tumor cell subclusters across different glioma grades. (D) Dot plot annotation of macrophage subcluster markers in IDH‐mutant gliomas. (E) Bar plot showing proportions of macrophage subclusters in IDH‐mutant gliomas. (F) Violin plots of key crosstalk markers in the VEGF pathway. (G) Dot plot of key crosstalk markers in the TGF‐β pathway. (H‐I) SPOTLIGHT deconvolution feature plot of the MES‐like and endothelial cells in spatial transcriptomics. [file CNS-31-e70371-s002.tif]
